# Predicting intraocular pressure using systemic variables or fundus photography with deep learning in a health examination cohort

**DOI:** 10.1038/s41598-020-80839-4

**Published:** 2021-02-11

**Authors:** Kaori Ishii, Ryo Asaoka, Takashi Omoto, Shingo Mitaki, Yuri Fujino, Hiroshi Murata, Keiichi Onoda, Atsushi Nagai, Shuhei Yamaguchi, Akira Obana, Masaki Tanito

**Affiliations:** 1 Department of Ophthalmology, Seirei Hamamatsu General Hospital, Hamamatsu, Shizuoka Japan; 2 Seirei Christopher University, Hamamatsu, Shizuoka Japan; 3 Department of Ophthalmology, The University of Tokyo, Tokyo, Japan; 4 Department of Neurology, Shimane University Faculty of Medicine, Izumo, Japan; 5 Department of Ophthalmology, Shimane University Faculty of Medicine, Izumo, Japan; 6 Faculty of Psychology, Outemon Gakuin University, Osaka, Japan; 7 Hamamatsu BioPhotonics Innovation Chair, Institute for Medical Photonics Research, Preeminent Medical Photonics Education & Research Center, Hamamatsu University School of Medicine, Hamamatsu, Shizuoka Japan

**Keywords:** Translational research, Medical research

## Abstract

The purpose of the current study was to predict intraocular pressure (IOP) using color fundus photography with a deep learning (DL) model, or, systemic variables with a multivariate linear regression model (MLM), along with least absolute shrinkage and selection operator regression (LASSO), support vector machine (SVM), and Random Forest: (RF). Training dataset included 3883 examinations from 3883 eyes of 1945 subjects and testing dataset 289 examinations from 289 eyes from 146 subjects. With the training dataset, MLM was constructed to predict IOP using 35 systemic variables and 25 blood measurements. A DL model was developed to predict IOP from color fundus photographs. The prediction accuracy of each model was evaluated through the absolute error and the marginal R-squared (mR^2^), using the testing dataset. The mean absolute error with MLM was 2.29 mmHg, which was significantly smaller than that with DL (2.70 dB). The mR^2^ with MLM was 0.15, whereas that with DL was 0.0066. The mean absolute error (between 2.24 and 2.30 mmHg) and mR^2^ (between 0.11 and 0.15) with LASSO, SVM and RF were similar to or poorer than MLM. A DL model to predict IOP using color fundus photography proved far less accurate than MLM using systemic variables.

## Introduction

Intraocular pressure (IOP) is a measure of the fluid pressure within the eye and it is an important marker for many ophthalmological diseases, including glaucoma, which is one of the world’s leading causes of irreversible blindness^1^. IOP is the result of the balance between the rates of aqueous humor production at the ciliary body and aqueous outflow from the eye through the conventional and uveoscleral pathways. The magnitude of IOP is primarily decided by local factors, such as resistance of the trabecular meshwork and juxtacanalicular connective tissues^2–4^. However, in the conventional pathway, aqueous humor is drained into the Schlemm’s canal and ultimately the episcleral vein^2^, and thus IOP is also affected by exogenous (systemic) factors, as suggested by a recent study^5^. Indeed, we recently investigated the associations of various systemic factors with IOP using a dataset from a health examination program database, and it was suggested that some of these were significantly associated with IOP level, including age, percent body fat, systolic blood pressure (SBP), pulse rate, albumin, and hemoglobin A1c (HbA1c)^3^. The first purpose of the current study was to investigate how much of IOP can be explained using various systemic factors.

It would be beneficial to predict IOP accurately using only systemic factors and without a tonometry at various settings, such as medical check-up, however it is presumed that IOP is not only decided by systemic factors, but also local (ocular) conditions. A fundus photography is one of the most representative and basic ophthalmological measurement. There have been remarkable recent developments in artificial intelligence (AI) and its application to a fundus photography. For instance, Poplin et al. showed that the sex of an individual can be identified from a color fundus photograph using DL with 97% accuracy^6^. We have also reported that an accurate diagnosis of glaucoma can be achieved, using a similar number of fundus photographs (3132 images) with the current study^7–9^, similar to other recent studies^10–16^. These results could imply that useful ophthalmological information can be extracted from a color fundus photograph using DL. The second purpose of the current study was to investigate the accuracy of predicting IOP using fundus photography and deep learning (DL).

## Methods

### Subjects

The Institutional Review Board of the Shimane University Faculty of Medicine approved this study (IRB No. 20190131-1), which was conducted according to the tenets of the Declaration of Helsinki. Each participant provided Informed Consent. The cohort database included 6272 examinations from 2577 subjects who participated in a health examination system in the Shimane Institute of Health Science^17,18^ from August 3, 1998, to March 28, 2019. We chose 6519 examinations from 5645 eyes of 2835 subjects from the database who had a complete measurements of: age, sex, height, body mass index (BMI), systolic blood pressure (SBP), diastolic blood pressure (DBP), history of diabetes mellitus (DM), history of hypertension (HT), history of hyperlipidemia, past and current smoking habitat, 25 blood examinations (total protein (TP), albumin/globulin ratio (A/G), aspartate aminotransferase (AST), alanine aminotransferase (ALT), guanosine triphosphate (γGTP), alkaline phosphatase (ALP), total cholesterol (TC), triglyceride (TG), high-density lipoprotein cholesterol (HDL-C), low-density lipoprotein cholesterol (LDL-C), hemoglobin A1c (HbA1c), white blood cell (WBC) count, red blood cell (RBC) count, hemoglobin (Hb), hematocrit (Ht), platelet (Plt) count, fibrinogen, blood urea nitrogen (BUN), creatinine (Cre), sodium (Na), potassium (K), chlorine (Cl), calcium (Ca), uric acid (UA), and amylase), IOP, and a color fundus photograph. BMI was calculated as body weight (kg) divided by the square of the body height (m). Experienced laboratory technicians measured IOP using a non-contact tonometer (Full Auto Tonometer TX-F, Canon Incorporated, Tokyo, Japan). Color fundus photographs were obtained using a non-mydriatic fundus camera with a 45 view-angle (before December 2012 using CR6-45NM, Canon, Tokyo, Japan, and after January 2013 using CR-2, Canon).

### Training and validation datasets

All of the measurements obtained by December 31, 2016 were assigned to the training dataset, which consisted of 3883 examinations from 3883 eyes of 1945 subjects. A validation dataset was also prepared for the purpose of DL parameter tuning, using data obtained between January 1, 2017 and December 31, 2017 (454 examinations from 454 eyes from 229 subjects).

### Testing dataset

The testing dataset was prepared using data obtained between January 1, 2017 and December 31, 2017 (289 examinations from 289 eyes from 146 subjects). There was no overlap among the three datasets.

### DL model to predict IOP from fundus photography

We adopted a type of convolutional neural network (CNN) known as ResNet^6^ to predict IOP from fundus photographs, following our previous studies in which a diagnosis of glaucoma was predicted from fundus photographs^7,8,19^. Unlike the simple CNN, ‘identical skip connections’ that skip one or more layers are used in ResNet and features are propagated to succeeding layers, which is well-known to be useful for image classification and feature extraction. This is because it enables ResNet to facilitate a deeper and larger network, which is helpful to acquire more effective and conceptual features without overfitting. In the current study, a ResNet model with 18 layers was pre-trained with the ImageNet classification^20^. This methodology is inspired by recent successes in fine-tuning deep neural networks^21^, whereby parameters of a network are first derived in a different but large pre-training dataset and then used to initialize training in a new and smaller training dataset. We attempted further improvements of the model by applying image augmentation of the training data^22^: all of the images in the training; dataset were horizontally flipped. The last fully-connected layer in ResNet was used to output the predicted value of IOP. Left eyes were mirror imaged to right eyes. Details of the parameters used in ResNet were: learning rate: 0.01, batch size: 100, damping capacity: 0.9 and weight decay: 0.0001.

### Models to predict IOP from systemic variables

First, using the training dataset, a multivariate linear regression model (MLM) was built to predict IOP using 35 variables (age, sex, height, BMI, SBP, DBP, history of DM, history of HT, history of hyperlipidemia, past and current smoking habitat, 25 blood examinations). Using this model, IOP values in the testing dataset were predicted, and the absolute prediction error was calculated. A number of other prediction models were also constructed using the following machine learning methods: (1) support vector machine (SVM)^23^, (2) Random Forest (RF)^24^, and (3) least absolute shrinkage and selection operator regression (LASSO)^25,26^. Support vector machine performs regression in a latent space (kernel space) to yield an accurate prediction even in a non-linear regression. Random Forest consists of many decision trees (regression trees), and outputs the averaged value from all individual trees. Each tree is constructed using a different bootstrap sample from the original data (bootstrapping is repeated sampling until the original sample size is reached, allowing duplication). In LASSO, the sum of the absolute values of the regression coefficients is constrained or penalized, so that the final model gives an accurate prediction. The details of each method follow.

1. Support vector machine: radial basis function, penalty parameter = 1.0
2. Random forest: number of trees = 10,000, criterion = Gini index, minimum number of samples required to split an internal node = 2, the minimum number of samples required to be at a leaf node = 1
3. LASSO: optimum lambda value was decided the minimum prediction error with the leave-one cross validation within the training dataset.

Subsequently, using these models, IOP values in the testing dataset were predicted, and absolute prediction errors were calculated.

### Statistical analysis

Absolute prediction errors were compared using the linear mixed model whereby values were nested within patients. The linear mixed model adjusts for the hierarchical structure of the data, modeling in a way in which measurements are grouped within subjects to reduce the possible bias derived from the nested structure of data^27,28^.

Furthermore, the association between the predicted IOP values and actual IOP values in the testing dataset was calculated using the correlation coefficient. Again, considering the nested structure of the current dataset, the association was also calculated using the marginal R-squared (mR^2^) value with the linear mixed model, following a method proposed by Nakagawa and Holger^29^.

## Results

The characteristics of the 1569 study subjects (819 men, 52%; 750 women, 48%; mean age, 62.2 ± 8.7 years; range 27–92 years) are summarized in Table 1. The mean IOP was 12.8 ± 3.0 mmHg (range 7–33.1 mmHg) in the right eye and 12.8 ± 3.0 mmHg (range 7–33.8 mmHg) in the left eye.

**Table 1:** Subjects' demographic data.

Parameters	Training data	Testing data
	Mean ± SD	Mean ± SD
IOP (mmHg)	12.8 ± 3.1	12.3 ± 2.9
Age (years)	62.9 ± 9.3	62.7 ± 12.2
Male/female	1027/918	92/54
Height (cm)	159.6 ± 9.0	162.5 ± 9.1
BMI	23.1 ± 3.1	23.4 ± 3.2
SBP (mmHg)	129.7 ± 17.3	126.4 ± 18.4
DBP (mmHg)	73.9 ± 11.4	74.8 ± 11.6
Smoking habitat (current/past/none)	1181/273/491	80/23/43
TP (g/dl)	7.4 ± 0.4	7.5 ± 0.4
Albumin (g/dl)	4.4 ± 0.2	2.6–5.2
A/G	1.5 ± 0.2	0.9–4.6
AST (IU/l)	25.4 ± 12.5	24.8 ± 8.4
ALT (IU/l)	23.8 ± 15.8	24.4 ± 12.3
γGTP (IU/l)	43.4 ± 62.4	40.8 ± 39.8
ALP (IU/l)	224.9 ± 68.9	217.1 ± 64.0
TC (mg/dl)	210.2 ± 31.7	216.5 ± 36.7
TG (mg/dl)	116.9 ± 71.2	137.9 ± 135.7
HDL-C (mg/dl)	63.0 ± 16.2	63.8 ± 17.4
LDL-C (mg/dl)	121.6 ± 29.3	124.5 ± 32.3
HbA1c (%)	5.5 ± 0.7	5.9 ± 0.7
WBC (× 10^2^/μl)	5811.9 ± 1531.7	5445.8 ± 1562.3
RBC (× 10^4^/μl)	460.7 ± 41.3	468.3 ± 50.3
Hb (g/dl)	14.3 ± 1.4	14.6 ± 1.6
Ht (%)	42.8 ± 3.6	43.3 ± 4.2
Plt (× 10^4^/μl)	22.3 ± 3.5	23.0 ± 5.3
Fibrinogen (mg/dl)	290.5 ± 58.1	335.3 ± 67.0
BUN (mg/dl)	14.9 ± 3.8	15.1 ± 4.2
Cre (mg/dl)	0.7 ± 0.2	0.8 ± 0.2
Na (mEq/l)	141.9 ± 2.0	141.3 ± 2.0
K (mEq/l)	4.1 ± 0.3	4.2 ± 0.3
Cl (mEq/l)	103.2 ± 2.4	103.9 ± 2.3
Ca (mg/dl)	9.3 ± 0.3	9.4 ± 0.3
UA (mg/dl)	5.3 ± 1.3	5.4 ± 1.2
Amylase (IU/l)	82.0 ± 27.6	80.9 ± 29.2

*IOP* intraocular pressure, *SD* standard deviation, *BMI* body mass index, *SBP* systolic blood pressure, *DBP* diastolic blood pressure, *TP* total protein, *A/G* albumin/globulin, *AST* aspartate aminotransferase, *ALT* alanine aminotransferase, *γGTP* guanosine triphosphate, *ALP* alkaline phosphatase, *HDL-C* high-density lipoprotein cholesterol, *LDL-C* low-density lipoprotein cholesterol, *HbA1c* glycosylated hemoglobin A1c, *WBC* white blood cell, *RBC* red blood cell, *BUN* blood urea nitrogen, *Na* sodium, *k* potassium, *Cl* chlorine, *Ca* calcium.

The results of univariate analyses between various systemic parameters and the IOP are summarized in Table 2. Among 35 parameters, 28 parameters showed significant association with IOP when not adjusted for age and sex (p < 0.05). When adjusted for age and sex, 23 (among 33) parameters showed significant association with IOP.

**Table 2:** Results of univariate analyses between IOP and various systemic parameters.

	Correlation coefficient	p value^†^	p value^‡^
Age	− 0.13	< 0.001*	–
Height	− 0.029	0.071	< 0.001*
Sex	− 0.037	0.022*	–
BMI	0.11	< 0.001*	< 0.001*
DM	0.033	0.039*	0.0011*
Hyperlipidemia	0.027	0.089	0.13
SBP	0.15	< 0.001*	< 0.001*
DBP	0.14	< 0.001*	< 0.001*
HT	0.047	0.0034*	< 0.001*
Smoking habitat	0.015	0.35	< 0.001*
TP	0.088	< 0.001*	< 0.001*
A/G	0.048	0.0030*	0.13
AST	0.060	< 0.001*	< 0.001*
ALT	0.10	< 0.001*	< 0.001*
γGTP	0.091	< 0.001*	< 0.001*
ALP	0.052	0.0013*	< 0.001*
TC	0.049	0.0024*	0.064
TG	0.096	< 0.001*	< 0.001*
HDL-C	− 0.013	0.43	0.19
LDL-C	0.036	0.024*	0.27
HbA1c	0.096	< 0.001*	< 0.001*
WBC	0.081	< 0.001*	< 0.001*
RBC	0.14	< 0.001*	< 0.001*
Hb	0.13	< 0.001*	< 0.001*
Ht	0.13	< 0.001*	< 0.001*
Plt	0.060	< 0.001*	0.066
Fibrinogen	− 0.017	0.92	0.23
BUN	− 0.068	< 0.001*	0.012*
Cre	− 0.052	0.0011*	0.046*
Na	0.018	0.27	0.97
K	− 0.020	0.22	0.61
Cl	− 0.036	0.024*	< 0.001*
Ca	0.074	< 0.001*	< 0.001*
UA	0.033	0.037*	< 0.001*
Amylase	− 0.048	0.0029*	0.093

All analyses were performed using Pearson’s rank correlation test.

The asterisk (*) indicates p < 0.05.

p value^†^: without adjustment for age, p value^‡^: with adjustment for age.

*IOP* intraocular pressure, *BMI* body mass index, *BP* blood pressure, *BNP* brain natriuretic peptide, *TP* total protein, *A/G* albumin/globulin, *AST* aspartate aminotransferase, *ALT* alanine aminotransferase, *γGTP* guanosine triphosphate, *ALP* alkaline phosphatase, *HDL-C* high-density lipoprotein cholesterol, *LDL-C* low-density lipoprotein cholesterol, *HBA1c* glycosylated hemoglobin A1c, *WBC* white blood cell, *RBC* red blood cell, *BUN* blood urea nitrogen, *Na* sodium, *K* potassium, *Cl* chlorine, *Ca* calcium.

The absolute prediction error with each method is shown in Table 3.

**Table 3:** The absolute prediction error with each method.

	Absolute prediction error Mean ± SD (dB)	p value
MLM	2.29 ± 1.5	–
LASSO	2.29 ± 1.5	0.80
SVM	2.24 ± 1.5	0.080
RF	2.30 ± 1.6	0.75
DL with color fundus photograph	2.70 ± 2.1	0.019

p value was calculated against MLM.

*SD* standard deviation, *MLM* multivariate linear regression, *LASSO* least absolute shrinkage and selection operator regression, *SVM* support vector machine, *RF* random forest, *DL* deep learning.

Table 4 shows the results of the MLM obtained with the training dataset. Among the 35 parameters, 11 showed a significant association with IOP p < 0.05), including Height, BMI, Age, sex, smoking habitat, TP, HbA1c and SBP.

**Table 4:** Result of MLM obtained with the training dataset.

	Coefficient	Standard error	p value
Age (years)	− 0.044	0.0067	< 0.001*
Height (cm)	− 0.030	0.0088	< 0.001*
Sex	− 0.85	0.20	< 0.001*
BMI	0.039	0.019	0.037*
DM	0.0079	0.19	0.97
Hyperlipidemia	− 0.080	0.10	0.43
SBP (mmHg)	0.026	0.0045	< 0.001*
DBP (mmHg)	0.0028	0.0067	0.67
HT	− 0.20	0.11	0.084
Tobacco	0.24	0.074	0.0014*
TP (g/dl)	0.69	0.17	< 0.001*
A/G	1.36	0.30	< 0.001*
AST (IU/l)	< 0.001	0.0072	0.89
ALT (IU/l)	0.0019	0.0055	0.74
γGTP(IU/l)	0.0026	0.0010	0.0089*
ALP (IU/l)	< 0.001	< 0.001	0.96
TC (mg/dl)	− 0.011	0.0067	0.099
TG (mg/dl)	0.0025	0.0014	0.078
HDL-C (mg/dl)	0.017	0.0076	0.029*
LDL-C (mg/dl)	0.0097	0.0066	0.14
HbA1c (%)	0.44	0.087	< 0.001*
WBC (× 10^2^/μl)	< 0.001	< 0.001	0.36
RBC (× 10^4^/μl)	0.0012	0.0023	0.59
Hb (g/dl)	0.20	0.10	0.062
Ht (%)	0.0086	0.047	0.85
Plt (× 10^4^/μl)	0.010	0.010	0.31
Fibrinogen (mg/dl)	< 0.001	< 0.001	0.93
BUN (mg/dl)	− 0.017	0.015	0.24
Cre (mg/dl)	− 0.33	0.36	0.36
Na (mEq/l)	− 0.0022	0.032	0.94
K (mEq/l)	− 0.21	0.15	0.18
Cl (mEq/l)	− 0.072	0.027	0.79
Ca (mg/dl)	− 0.25	0.18	0.17
UA (mg/dl)	0.088	0.048	0.069
Amylase (IU/l)	0.0024	0.0019	0.20

*Represents the p value < 0.05.

*MLM* multivariate linear regression, *IOP* intraocular pressure, *BMI* body mass index, *BP* blood pressure, *BNP* brain natriuretic peptide, *TP* total protein, *A/G* albumin/globulin, *AST* aspartate aminotransferase, *ALT* alanine aminotransferase, *γGTP* guanosine triphosphate, *ALP* alkaline phosphatase, *HDL-C* high-density lipoprotein cholesterol, *LDL-C* low-density lipoprotein cholesterol, *HBA1c* glycosylated hemoglobin A1c, *WBC* white blood cell, *RBC* red blood cell, *BUN* blood urea nitrogen, *Na* sodium, *k* potassium, *Cl* chlorine, *Ca* calcium.

**Figure 1:**
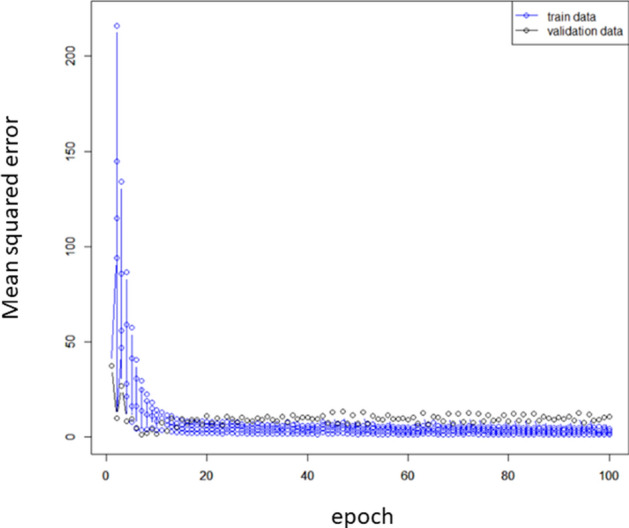
The mean squared error, of the DL model, with the validation dataset at each epoch. The mean squared error saturated at < 100 epochs.

**Figure 2:**
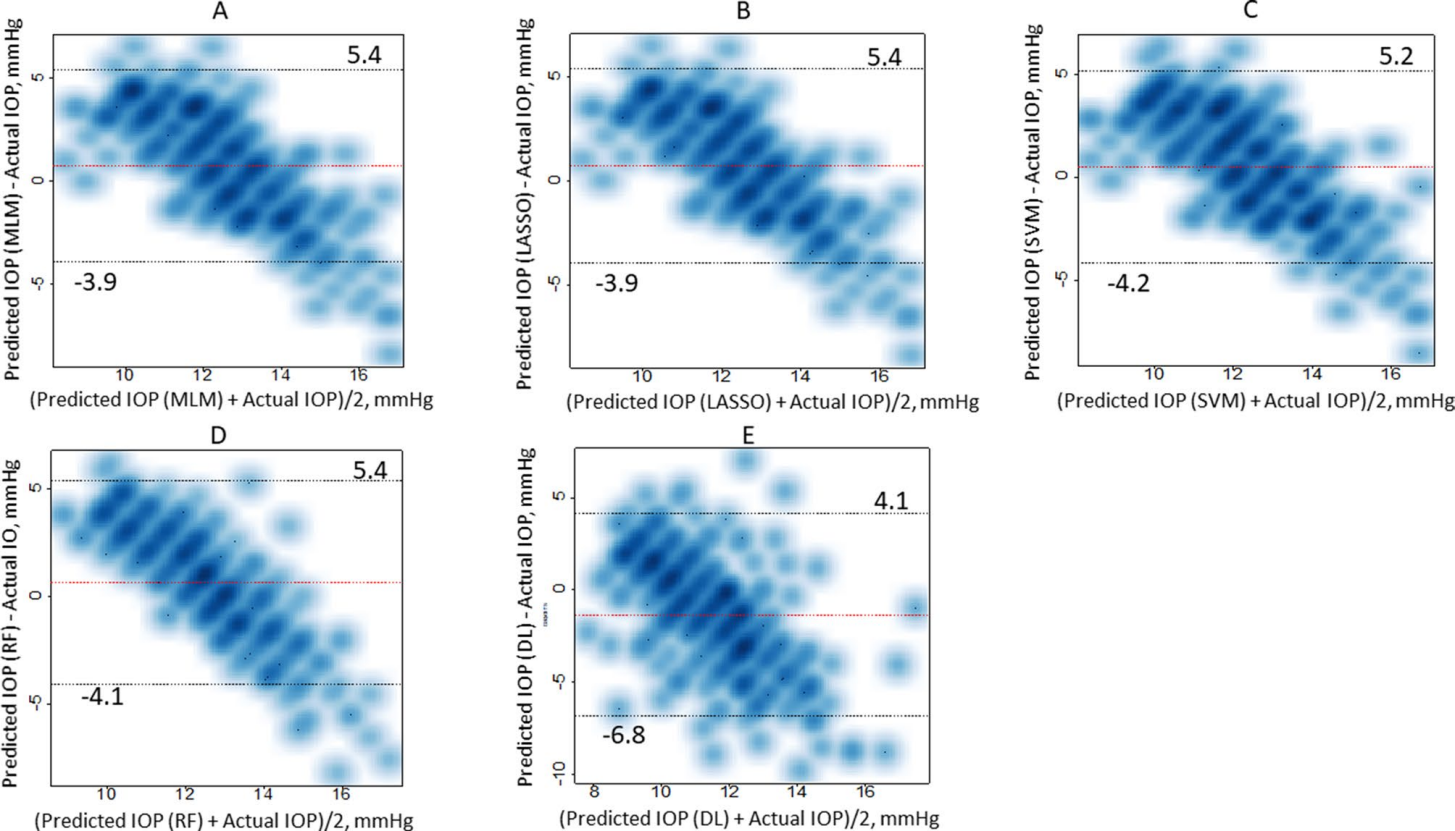
The relation between the predicted IOP values with each prediction method and actual IOP value, shown as a Bland–Altman plot. (**a**) MLM, (**b**) LASSO, (**c**) SVM, (**d**) RF, (**e**) DL. Data was shown as a smoothed scatter plot. *MLM* multivariate linear regression, *LASSO* least absolute shrinkage and selection operator regression, *SVM* support vector machine, *RF* random forest, *DL* deep learning.

The mean squared error, for the DL model, with the validation dataset saturated at < 100 epochs, as shown in Fig. 1. The predicted IOP values were derived from epoch = 100. The relationship between the predicted IOP values with each prediction method and actual IOP value is shown in Fig. 2a–e, using the Bland Altman plot. The correlation coefficient and mR^2^ values of these variables are shown in Table 5. Significant correlations were observed between IOP and the predicted IOP values with MLM, LASSO, SVM, and RF (p < 0.05), but not with the DL model using color fundus photographs (p = 0.16 or 0.17). There was a significant association between (difference between predicted IOP and actual IOP) and (mean of predicted IOP and actual IOP) with all models (p < 0.001).

**Table 5:** The correlation coefficient and mR^2^ values of these variables.

	Correlation coefficient	p value	mR^2^	p value
MLM	0.38	< 0.001	0.15	< 0.001
LASSO	0.38	< 0.001	0.15	< 0.001
SVM	0.38	< 0.001	0.15	< 0.001
RF	0.34	< 0.001	0.11	< 0.001
DL with color fundus photograph	0.083	0.16	0.0066	0.17

*mR*^*2*^ marginal R-squared value (following a method proposed by Nakagawa and Holger^29^), *MLM* multivariate linear regression, *LASSO* least absolute shrinkage and selection operator regression, *SVM* support vector machine, *RF* random forest, *DL* deep learning.

**Figure 3:**
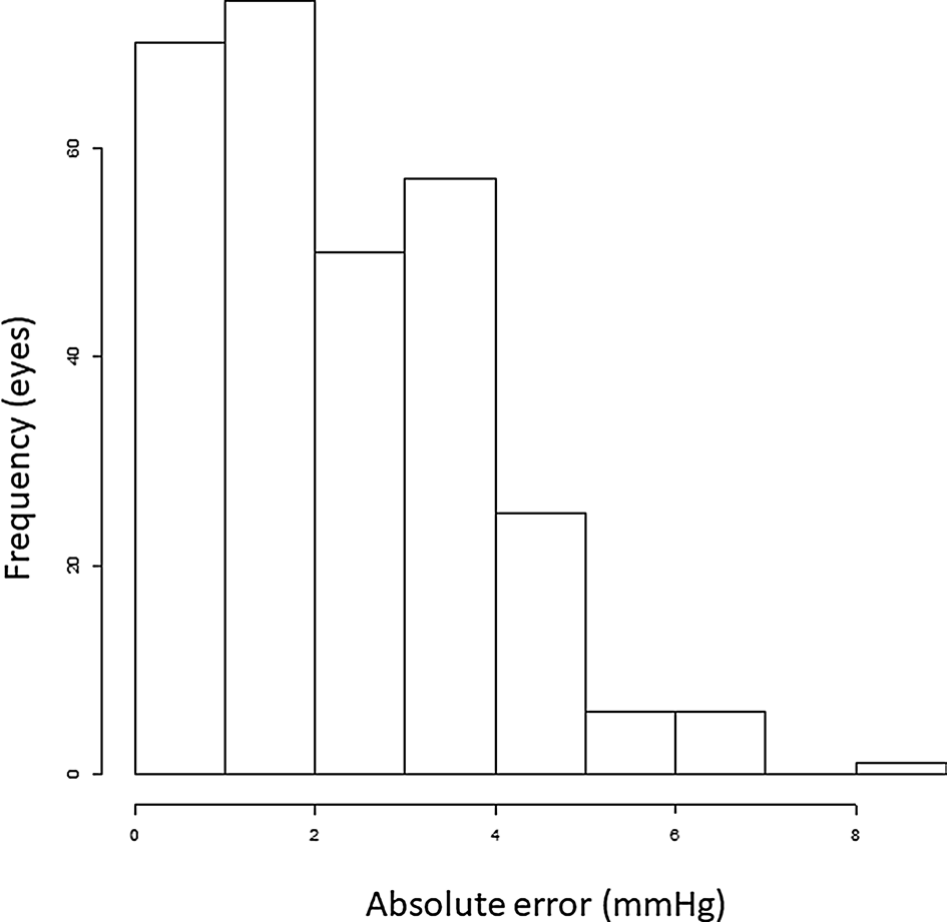
Histogram of absolute prediction error associated with MLM model. *MLM* multivariate linear regression.

The absolute error associated with MLM is illustrated in Fig. 3.

## Discussion

In the current study, IOP was predicted using a variety of modelling methods and different data. A considerably more accurate prediction of IOP was achieved using a MLM of systemic variables (mean absolute error = 2.29 dB and mR^2^ = 0.15) compared to a DL model with color fundus photography (mean absolute error = 2.70 dB and mR^2^ = 0.0066). Machine learning methods (LASSO, SVM and RF) did not improve prediction accuracy.

The MLM included 11 variables that were significantly correlated with IOP. We recently reported that several systemic factors were associated with IOP level, including age, percent body fat, SBP, pulse rate, albumin, and HbA1c^30^. We observe that older age, higher SBP, and higher HbA1c were again significantly associated with increased IOP. The effect of age on IOP is controversial. Previous cross-sectional studies from Italy^31^ and the United States^32,33^ suggested a significant positive association between age and IOP, however, the inverse effect has also been reported in cross-sectional or longitudinal studies from other countries, mainly in Asia, including Japan^34–39^. The current study—conducted in Japan—also suggested a negative association between age and IOP. The significant positive correlation between higher SBP and IOP is in agreement with other previous studies^33,35,37–46^, where the mechanism has been speculated as an increased filtration fraction of the aqueous humor through elevated ciliary artery pressure, increased serum corticoids and also sympathetic tone result in elevated IOP^47,48^. The association between HbA1c and IOP is also in agreement with previous studies^33–35,37,39,42–44,46,47,49,50^. Several mechanisms have been reported for obesity to be associated with increasing IOP, such as sympathetic hyperactivation, increased corticosteroid, excessive intraorbital adipose tissue, increases in blood viscosity with high hemoglobin and hematocrit values, increased episcleral venous pressure, a consequent decrease in the facility of aqueous outflow also transitory elevations in IOP resulting from breath-holding and thorax compression while tonometry is performed during slit-lamp examinations in obese patients^47,51–54^. Our previous study suggested percent body fat is associated with increased IOP, whereas this was the case for BMI in the current study. Smoking status was significantly associated with elevated IOP, agreeing with a previous study^55^.

It is widely acknowledged that ordinary statistical models, such as linear or binomial logistic regression, may be over-fitted to the original sample, especially when the number of predictor variables is large. We have reported on the usefulness of applying machine learning methods for many applications, including diagnosing glaucoma from optical coherence tomography measurements^56–59^, predicting vision related quality of life^60^, and VF progression^61–63^, compared to ordinal linear or logistic regression. Nonetheless, in the current study, there was no improvement in the prediction accuracy of machine learning methods compared to the MLM. This may be because of the size of the training dataset was quite large (5540 examinations) and therefore overfitting was less of a problem. Despite the significant association between predicted IOP and true IOP, only a moderate mR^2^ value was obtained (up to 0.15). Coefficient of determination value represents how much of the data is explained by the model. Correlation coefficient is identical to the square root of coefficient of determination value. The mR^2^ value shows how much of the data can be explained by the fixed effect in the linear mixed model. Hence, the current results suggested that approximately 15% of IOP was explained by MLM and other machine learning models. In other words, our results suggested IOP can be only partially explained by systemic factors, and the remaining part may only be described locally (using measurements from the eye). As shown in the Bland–Altman plots (Fig. 2), the distribution of the difference between the predicted and actual IOP values were not horizontal, and correlated with the mean of these values. This is because the prediction accuracy was relatively poor and the predicted values were relatively constant regardless of the actual IOP value. Furthermore, although it has been suggested that the Random Forests method is more useful than other machine learning methods^64–66^, this merit was not observed compared to other machine learning methods in the current study. These finding would also support that IOP can be only partially explained by systemic factors, and the predictability cannot be considerably improved by merely applying machine leaning methods, such as the Random Forests.

A recent study revealed that DL could discriminate sex from fundus photography with very high accuracy^6^. In contrast, we recently suggested that the discrimination of sex can be achieved, at least to some extent (AUC = 77.9%), using a ‘visible’ machine learning method (LASSO) with clinically meaningful variables such as color intensities, tessellation, and also geometrical information of the optic disc and retinal vessels. As a result, it was implied that the DL model learned a principle to discriminate sex from color fundus photographs. On the other hand, the current study suggested that DL was not accurate to predict IOP from fundus photographs since there only a poor association (mR^2^ = 0.0066) was observed between the IOP predicted from this approach and actual IOP. We attempted other DL methods, instead of ResNet18 (VGG16^67^ and Inception-v3^68^), however, results were not improved (data not shown in “Result”). This may suggest little valuable information is present in color fundus photography regarding IOP. This study included a fairly large training dataset, however, it was much smaller compared to other representative datasets for DL, such as ImageNet (14,000,000 images)^20^ and CIFAR10 (60,000 images, https://www.cs.toronto.edu/~kriz/cifar.html), although we have recently suggested the diagnosis of glaucoma, using color fundus photographs and DL, can be achieved with an even smaller sample size (N = 3132)^7–9^. Better results might be observed if DL was applied to a larger dataset. The current results suggested that IOP can only be partially explained using systemic factors (15%; as suggested by the mR^2^ value) or color fundus photography with DL (0.66%), which implies we need to continue to conduct IOP measurement using a tonometry. The merit of accurately predicting systemic factors using a color fundus photograph, such as shown in^69^, cannot be overestimated, such as medical check up in developing countries without tonometry. This is in particular true with a smart-phone base fundus photography, since recent studies have suggested that the usefulness of a deep learning-assisted program to screen for retinal diseases using a smartphone^70,71^.

The current study had several limitations, the first of which was the use of non-contact tonometry, which is generally believed to be less reliable than Goldmann applanation tonometry (the repeatability coefficient with non-contact tonometry has been reported as ± 3.2 mmHg, whereas that with Goldmann applanation tonometry was between ± 2.2 and 2.5 mmHg)^72,73^ although IOP is usually measured using the non-contact tonometry in a health examination outside eye clinics. Further, there was an absence of central corneal thickness measurements that are known to induce measurement errors during tonometry^74,75^ In addition, the usefulness of applying DL to color fundus photography in glaucomatous eyes should also be investigated in a future study. The current study consisted of a health examination cohort, and hence the vast majority cases had normal IOP values. A further study is needed to investigate whether the current approach is more useful in eyes with higher IOP values. In particular it should be further investigated that whether DL enables more accurate prediction of IOP using a larger dataset.

In conclusion, the current study, using a health examination cohort, suggested that IOP cannot be adequately predicted from clinical parameters or retinal photographs even using state-of-art ML techniques. Further investigation with DL using a larger amount of data would be needed.
